# Late-onset *Exophiala dermatitidis* keratitis after cataract surgery: A case report and literature review

**DOI:** 10.1016/j.mmcr.2026.100805

**Published:** 2026-06-03

**Authors:** Atsuhiko Fukuto, Suzu Deie, Yosuke Inada, Takashi Yaguchi, Hirokazu Sakaguchi, Taiichiro Chikama

**Affiliations:** a Department of Ophthalmology and Visual Sciences, Graduate School of Biomedical and Health Sciences, Hiroshima University, Hiroshima, 734-8551, Japan; b Medical Mycology Research Center, Chiba University, Chiba, 260-8673, Japan

**Keywords:** *Exophiala dermatitidis*, Fungal keratitis, Dematiaceous fungi, Cataract surgery, Penetrating keratoplasty

## Abstract

We report *Exophiala dermatitidis* keratitis developing one year following cataract surgery in a 73-year-old immunocompetent man. Before the correct diagnosis was established, the patient was initially treated for suspected epithelial ingrowth with corticosteroids for ten months. Therapeutic penetrating keratoplasty was required, despite intensive antifungal therapy including topical and systemic voriconazole and amphotericin B. This case underscores the criticality of considering fungal infection in atypical postoperative presentations with prolonged corticosteroid exposure.

## 1 Introduction

*Exophiala* species are dematiaceous fungi widely distributed in nature, mainly causing systemic infections in immunocompromised patients, with ocular infections being exceptionally rare [1,2]. Fungal keratitis is a significant clinical difficulty, with dematiaceous fungi including *Exophiala* species increasingly recognized as emerging pathogens [3]. These organisms generally require predisposing factors, such as ocular trauma, corneal surgery, or immunosuppression, for infection to occur [4]. Post-cataract surgery fungal infections are rarely reported, and using topical corticosteroids may predispose patients to opportunistic fungal infections [4].

Herein, we report a rare case of *E. dermatitidis* keratitis developing one year following uncomplicated cataract surgery in an immunocompetent patient. This study includes a comprehensive literature review of all 16 reported cases of *Exophiala* keratitis. This case shows the diagnostic and therapeutic difficulties of this uncommon infection and emphasizes the criticality of considering fungal etiology in atypical postoperative presentations, especially following prolonged corticosteroid therapy.

## 2 Case presentation

A 73-year-old Japanese man presented to our institution with progressive blurred vision in the left eye. The patient had undergone uneventful phacoemulsification with implantation of intraocular lens (IOL) in the left eye at a local ophthalmology clinic approximately one year earlier. The medical history was unremarkable, with no diabetes mellitus, immunosuppressive conditions, or systemic corticosteroid use reported. No history of ocular trauma or exposure to soil or organic materials was present.

One year following cataract surgery, the patient developed blurred vision in the left eye and sought assessment at a local clinic. Slit-lamp examination showed proliferative material at the 10 o'clock side-port incision, which was clinically suspected to represent an epithelial ingrowth. Topical betamethasone phosphate was initiated based on this presumed diagnosis. However, the patient underwent anterior chamber irrigation to remove the proliferative material after three months of corticosteroid therapy without improvement. Given that an infectious etiology was not suspected, no microbiological cultures were obtained at the time of the procedure.

Seven months following anterior chamber irrigation, the proliferative changes recurred and progressively worsened despite ongoing topical corticosteroid therapy. The patient was referred to the Department of Ophthalmology at Hiroshima University Hospital for further evaluation and management approximately 22 months after the original cataract surgery.

**Fig. 1:**
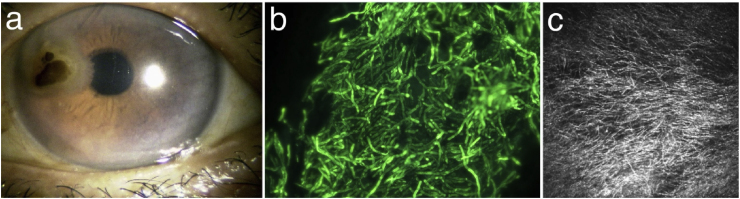
a) Slit-lamp photograph of the left eye at the initial presentation. A 4-mm brownish lesion was noted in the nasal cornea with surrounding feathery opacities extending into the stroma. No conjunctival injection was observed, and the anterior chamber revealed minimal inflammation. b) Fungiflora Y staining of the corneal scraping showing several septate hyphae, consistent with a filamentous fungal infection. c) *In vivo* confocal microscopy image showing highly reflective branching filamentous structures throughout the corneal stroma, consistent with the fungal hyphae of *Exophiala dermatitidis*.

At initial presentation to our institution, the patient's best-corrected visual acuity in the left eye was 20/40. Slit-lamp examination showed no significant inflammation in the anterior chamber. The cornea had a distinctive 4-mm brownish lesion in the nasal region, with surrounding feathery opacities extending into the stroma (Fig. 1a). The IOL was well positioned, without evidence of dislocation or opacification. The right eye examination was unremarkable, with normal vision.

Fungal keratitis was suspected, given the clinical appearance and chronic progressive course. Corneal scraping was performed for microbiological examinations. Fungiflora Y staining showed multiple septate hyphae (Fig. 1b). *In vivo* confocal microscopy showed highly reflective branching filamentous structures in the superficial stroma, confirming the presence of fungal elements (Fig. 1c). These results established a fungal keratitis diagnosis. The frequency of topical betamethasone phosphate application was decreased from four times a day to twice a day. Topical 5% natamycin was initiated four times daily along with 1% voriconazole eyedrops six times daily. Furthermore, systemic antifungal therapy was initiated with intravenous voriconazole (250 mg daily). The corneal scraping specimens were inoculated onto potato dextrose agar and incubated at 28 °C.

The culture yielded darkly pigmented colonies after 5 days of incubation. MALDI-TOF MS analysis of the isolated organism provided rapid identification as *Exophiala dermatitidis* with a high confidence score. Subsequently, this identification was confirmed by DNA sequencing of the internal transcribed spacer (ITS) region, which revealed 100% homology with *E. dermatitidis* reference strains in the GenBank database. The isolate was preserved as IFM 70175 at the Medical Mycology Research Center, Chiba University through the National Bio-Resource Project, Japan (JPMBRP-202228).

Antifungal susceptibility testing was conducted according to the Clinical and Laboratory Standards Institute (CLSI) M38-A2 guidelines for filamentous fungi. Reference powders of natamycin were obtained from FUJIFILM Wako Pure Chemical Corporation (Osaka, Japan), and those of all other antifungal agents tested from Eiken Chemical Co., Ltd. (Tokyo, Japan). Table 1 presents the minimum inhibitory concentrations (MICs). The isolate showed favorable susceptibility to azole antifungals, especially voriconazole (0.12 μg/mL) and itraconazole (0.25 μg/mL), and moderate susceptibility to amphotericin B (0.5 μg/mL) and natamycin (1 μg/mL).

**Table 1:** Susceptibility testing of the antifungal agents.

Antifungal agents	MIC (μg/mL)
Micafungin	8
Caspofungin	8
Amphotericin-B	0.5
Fluconazole	1
Flucytosine	8
Voriconazole	0.12
Itraconazole	0.25
Miconazole	0.25
Natamycin	1

MIC, minimum inhibitory concentration.

Minimal clinical improvement was noted after 3 weeks of intensive topical and systemic voriconazole therapy. Therefore, antifungal regimen was modified due to the slow therapeutic response and concerns regarding progressive stromal involvement. Topical natamycin was continued for a total of 4 weeks, whereas systemic therapy was switched to intravenous amphotericin B at 2.5 mg/kg daily. Additionally, topical therapy was changed to 0.1% amphotericin B eye drops, both administered for 2 weeks. However, the corneal infiltrate exhibited an insufficient response, despite this aggressive antifungal approach.

Thus, therapeutic penetrating keratoplasty (PKP) was performed 5 weeks after the initial presentation at our institution, given the inadequate response to medical therapy and the presence of deep stromal involvement. A 5-mm trephine was utilized to remove the infected corneal tissue, which was sent for histopathological examination. The excised corneal button was replaced with a donor cornea of the corresponding size obtained from the eye bank.

**Fig. 2:**
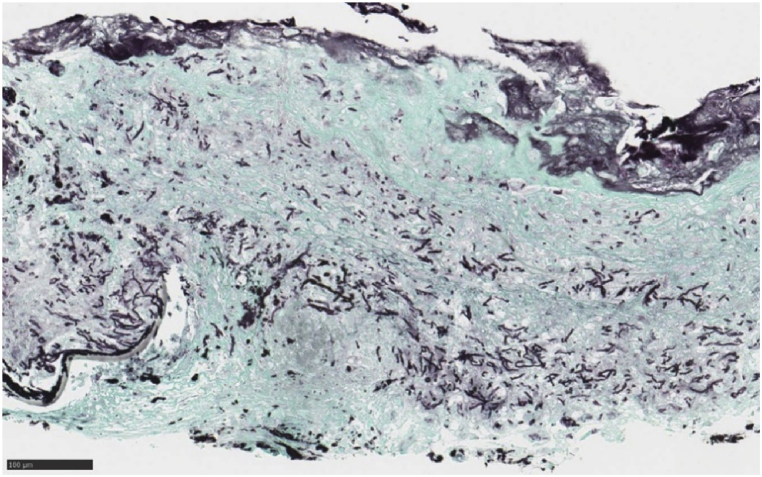
Histopathology of the excised corneal tissue. Grocott staining revealing septate, branching fungal hyphae extending through the full thickness of the stroma to the deep stromal layers, characteristic of dematiaceous fungi. The upper portion of the image represents the epithelial side, and the lower portion represents the endothelial side. Scale bar = 100 μm.

Histopathological examination of the excised corneal tissue using Grocott's methenamine silver stain showed fungal hyphae extending throughout the full thickness of the stroma, reaching the deep stromal layers (Fig. 2). The fungal elements appeared as septate, branching hyphae with characteristic pigmentation, consistent with a dematiaceous fungus.

Postoperatively, the patient received topical 1% voriconazole eye drops six times daily based on the susceptibility testing findings, which revealed excellent activity against the isolated strain. Notably, systemic antifungal therapy was not continued in the postoperative period because the infected tissue had been surgically removed, and no evidence of extension existed beyond the cornea. The surgical wound healed without complications.

**Fig. 3:**
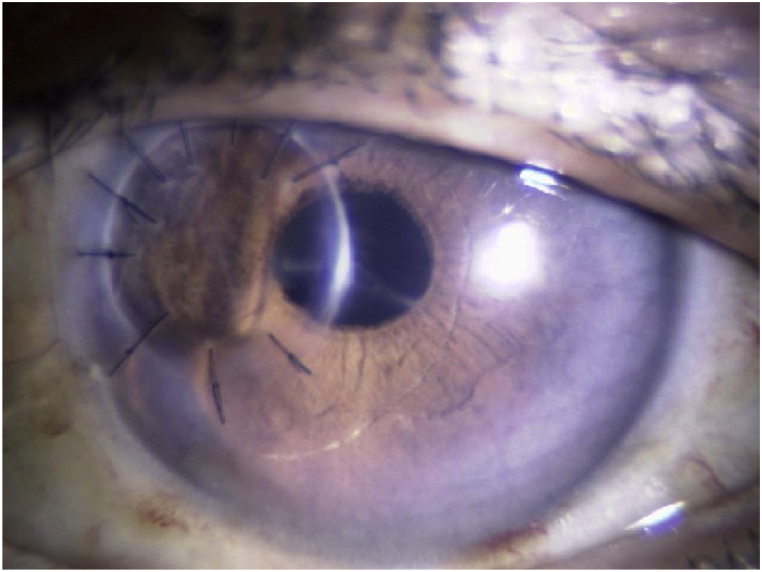
Slit-lamp photograph at 5 months postoperative follow-up revealing a clear corneal graft with well-opposed edges and no signs of infection recurrence or graft rejection. The anterior chamber was deep and quiet, and the intraocular lens remained well positioned.

The corneal graft remained clear at 5 months postoperatively, without signs of rejection or recurrent infection (Fig. 3). However, the patient's best-corrected visual acuity was 20/600 owing to substantial irregular astigmatism secondary to the graft. No recurrence of fungal infection was noted during the follow-up period. The patient continues to be regularly monitored for signs of graft failure or infection recurrence.

## 3 Discussion

This report presents a rare instance of *E. dermatitidis* keratitis occurring one year following cataract surgery in an immunocompetent patient. Unique aspects include delayed presentation, prolonged corticosteroid use after misdiagnosis as epithelial ingrowth, and the requirement for therapeutic keratoplasty despite intensive antifungal therapy. We performed a comprehensive literature review to place this case in the context of previously reported *Exophiala* keratitis cases.

A comprehensive review of the English literature identified 16 previously reported cases of *Exophiala* keratitis from 1990 to 2024 (Table 2). *E. dermatitidis* was the most reported species (6 cases), followed by *E. jeanselmei* (5 cases), and *E. phaeomuriformis* (3 cases). Furthermore, single cases each of *E. lecanii-corni* and *E. oligosperma* were identified. The age range was 25–84 years (mean 55.6 years). The geographic distribution revealed cases from North America (six cases), Europe (five cases), Asia (four cases), and Africa (one case). Notably, although India is among the largest global contributors to fungal and dematiaceous keratitis [3], no previously reported case of *Exophiala* keratitis from India was identified among the cases reviewed, underscoring the rarity of this organism as a corneal pathogen even in regions where fungal keratitis is endemic.

**Table 2:** Summary of the reported cases of *Exophiala* keratitis in the literature.

Species	Age/gender	Year	Region	Basic disease	Antifungal agent	Outcome	Reference
*E. dermatidis*	35/M	1990	Czechoslovakia	Recklinghausen's disease	Amphotericin B	Improved	[5]
	31/M	1998	Frace	PK, steroid	Amphotericin B, itraconazole	Therapeutic PK	[6]
	52/M	2006	USA	LASIK	Amphotericin B, Itraconazole, natamycin	Improved	[7]
	75/M	2006	Taiwan	PK, foreign body	Amphotericin B, fluconazole, itraconazole, natamycin	Therapeutic PK	[8]
	36/M	2019	Spain	KID syndrome, PK, CL	None	Therapeutic PK	[9]
	68/F	2023	Spain	PK	Voriconazole	Improved	[10]
*E. jeanselmei*	42/M	1993	Saudi Arabia	Unknown	Natamycin, miconazole	Improved	[11]
	58/F	2002	Israel	Trauma	Amphotericin B, natamycin	Improved	[12]
	39/F	2008	USA	LASIK, rock climbing	Amphotericin B, Itraconazole, natamycin	Improved	[13]
	41/M	2013	USA	Plant injury	Ketoconazole, natamycin, voriconazole	Improved	[14]
	25/M	2024	USA	LASIK	Natamycin	Improved	[15]
*E. lecanii-corni*	81/F	2020	Japan	DSAEK, steroid, CL	Natamycin, voriconazole	Improved	[16]
*E. oligosperma*	75/F	2021	South Africa	Cataract surgery, diabetes	Amphotericin B, voriconazole, natamycin	Evisceration	[17]
*E. phaeomuriformis*	84/F	2017	USA	PK, steroid	Amphotericin B, voriconazole	Improved	[18]
	67/M	2018	USA	KPro, steroid, CL	Amphotericin B	KPro removal and replacement	[19]
	81/F	2018	Sweden	CL	Amphotericin B, fluconazole	Improved	[20]

M, male; F, female; PK, penetrating keratoplasty; LASIK, laser in-situ keratomileusis; KID syndrome, Keratitis–ichthyosis–deafness syndrome; CL, contact lens; DSAEK, Descemet's stripping automated endothelial keratoplasty; KPro, keratoprosthesis.

The predisposing factors significantly varied among the reported cases. Previous corneal surgery, especially penetrating keratoplasty, was the most common predisposing factor (7 cases), followed by LASIK (4 cases). Previous cataract surgery was documented in only one case. Ocular trauma was identified in three cases, and contact lens wear was noted in four cases. Concomitant corticosteroid use was documented in six cases, underscoring the role of immunosuppression in enabling fungal proliferation.

The therapeutic methods differed across the reported cases. Topical antifungals were universally used, with amphotericin B being the most frequently used agent (12 cases), followed by natamycin (9 cases), voriconazole (5 cases), and itraconazole (6 cases). Combination therapy with multiple antifungal agents was common (12 cases). Systemic antifungal therapy was administered less frequently and was generally reserved for severe or recalcitrant infections.

Regarding outcomes, most patients exhibited clinical improvement with medical therapy alone (12 cases). However, four patients needed surgical intervention, including three therapeutic penetrating keratoplasties and one evisceration for *E. oligosperma* with progression to endophthalmitis after cataract surgery. Visual outcomes were generally favorable in patients who responded to medical therapy, although specific final visual acuity data were inconsistently reported across studies.

Our case shares multiple features with previously reported cases, including occurrence following ocular surgery and prolonged corticosteroid use, with the ultimate requirement for therapeutic keratoplasty. However, several features distinguish our case: the unusually delayed presentation one-year post-surgery, the extended period of corticosteroid therapy (10 months) before the correct diagnosis, and minimal clinical response despite favorable *in vitro* susceptibility and aggressive medical therapy. This case underscores the criticality of considering fungal infections in atypical postoperative presentations, especially when corticosteroid therapy fails to achieve improvement.

## Consent

Please declare that you have obtained written and signed consent to publish the case report from the patient or legal guardian(s).

## Please state that consent has been obtained from the patient or legal guardian(s)

Written informed consent was obtained from the patient or legal guardian(s) for publication of this case report and accompanying images. A copy of the written consent is available for review by the Editor-in-Chief of this journal on request.

## Funding source

All sources of funding should be acknowledged and you should declare any extra funding you have received for academic research of this work. If there are none state ‘there are none’.

## CRediT authorship contribution statement

**Atsuhiko Fukuto:** Conceptualization, Data curation, Formal analysis, Methodology, Project administration, Writing – original draft, Writing – review & editing. **Suzu Deie:** Investigation, Visualization, Writing – review & editing. **Yosuke Inada:** Investigation, Visualization, Writing – review & editing. **Takashi Yaguchi:** Formal analysis, Investigation, Methodology, Validation, Writing – review & editing. **Hirokazu Sakaguchi:** Resources, Supervision, Writing – review & editing. **Taiichiro Chikama:** Resources, Supervision, Validation, Writing – review & editing.

## Conflict of interest

Please declare any financial or personal interests that might be potentially viewed to influence the work presented. Interests could include consultancies, honoraria, patent ownership or other. If there are none state ‘there are none’.
